# Effectiveness of rehabilitation treatment for bone metastasis patients without surgical intervention: A propensity score matching analysis

**DOI:** 10.1016/j.jbo.2025.100703

**Published:** 2025-07-19

**Authors:** Ryo Yoshikawa, Yasumitsu Fujii, Ryoga Kashima, Wataru Saho, Risa Harada, Daisuke Makiura, Katsuya Fujiwara, Junichiro Inoue, Yoshiki Takeoka, Ryoko Sawada, Naomasa Fukase, Keisuke Oe, Hitomi Hara, Kenichiro Kakutani, Toshihiro Akisue, Yoshitada Sakai

**Affiliations:** a Kobe University Graduate School of Medicine, Division of Rehabilitation Medicine, Kobe, Hyogo, Japan; b Kobe University Hospital, Department of Physical Medicine and Rehabilitation, Kobe, Hyogo, Japan; c Kobe University Hospital, Department of Rehabilitation Medicine, Kobe, Hyogo, Japan; d Kobe University Graduate School of Health Sciences, Department of Rehabilitation Science, Kobe, Hyogo, Japan; e Kobe University Graduate School of Medicine, Department of Orthopaedic Surgery, Kobe, Hyogo, Japan

**Keywords:** Bone metastasis, Rehabilitation, Activities of daily living, Quality of life, Propensity score matching

## Abstract

Evidence regarding the effectiveness of rehabilitation treatments in patients with bone metastases remains limited. This study evaluated the implementation and effectiveness of rehabilitation in patients with bone metastases who did not undergo surgery. This retrospective study included 200 patients with nonsurgically treated bone metastases at our institution. The patients were categorized into a rehabilitation group (R group, n = 61) and a non-rehabilitation group (N group, n = 139). Over the course of one month, we compared activities of daily living (ADL), assessed using the Barthel Index (BI), quality of life (QOL), measured using the EuroQoL-5 Dimension (EQ-5D), and demographic and clinical characteristics. Propensity score matching was conducted to minimize selection bias. After matching, 31 patients in each group were included in the analysis. No statistically significant differences were observed in baseline BI and EQ-5D scores between the two groups. In the R group, BI improved significantly from 80 (interquartile range [IQR]: 60–100) to 90 (IQR: 70–100), and EQ-5D improved from 0.444 (IQR: 0.282–0.608) to 0.608 (IQR: 0.533–0.768). In contrast, no improvements were observed in either score in the N group. Chemotherapy was identified as a significant factor associated with improvements in BI (odds ratio 4.03) and EQ-5D (odds ratio 5.29). Rehabilitation may be a valuable treatment option for nonsurgically treated patients with bone metastases, warranting further validation in prospective studies.

## Highlights

• ADL and QOL were compared between bone metastasis patients with and without rehabilitation using propensity score matching.
• Rehabilitation improved ADL and QOL in patients with nonsurgically treated bone metastasis.
• Chemotherapy was associated with greater improvement in ADL and QOL.

## 1 Introduction

Advances in cancer treatment have considerably improved survival outcomes, leading to a marked increase in the number of patients surviving cancer. Bone metastasis is a common complication in patients with advanced cancers, with a prevalence of approximately 4.6 % among patients with solid tumors [1]. As the number of cancer survivors continues to increase, the incidence of bone metastases is also expected to increase further [1,2]. The treatment of metastatic bone diseases often does not focus on eradicating metastatic lesions but rather on improving or maintaining patients’ activities of daily living (ADL) and quality of life (QOL) [3].

Although cancer rehabilitation has been widely recommended by organizations such as the American Cancer Society and the American College of Sports Medicine for general cancer survivors [[4], [5], [6]], evidence regarding rehabilitation specifically for patients with bone metastases remains limited [7]. Patients with bone metastases present unique clinical challenges owing to skeletal fragility, pain, the risk of pathological fractures, and varying functional limitations, which often require careful modification of rehabilitation programs [3,8,9]. Moreover, while surgical interventions for bone metastases have been shown to improve ADL and QOL [10,11], many patients are not candidates for surgery because of their poor general condition, tumor burden, or other clinical factors. In nonsurgical patients, appropriately designed rehabilitation programs may still offer substantial benefits by helping preserve functional independence, preventing further deconditioning, and improving overall well-being. Despite these potential advantages, few studies have systematically evaluated the safety, feasibility, and effectiveness of rehabilitation interventions in this patient population [12].

In this study, we hypothesized that rehabilitation would improve ADL and QOL in patients with bone metastases who did not undergo surgery. Therefore, we investigated the status of rehabilitation in these patients. We performed a comparative analysis using propensity score matching to evaluate the effects of rehabilitation treatment. If this hypothesis is confirmed, then rehabilitation may be recommended as a valuable treatment option for patients with bone metastases.

## 2 Methods

### 2.1 Ethics

This study was approved by the Institutional Review Board of Kobe University Hospital (approval no. B210055). Written informed consent was obtained from all patients, in accordance with the principles outlined in the Declaration of Helsinki.

### 2.2 Participants and data collection

This retrospective cohort study enrolled 668 patients treated for bone metastases at Kobe University Hospital between January 2013 and December 2021. The analysis focused on the changes observed at the one-month follow-up and included only patients who completed the follow-up. Patients who underwent surgery during the study period were also excluded. In this study, surgical treatments for bone metastases were defined as bone cement augmentation, osteosynthesis, and joint replacement for extremity metastases as well as spinal decompression, instrumented fusion, and percutaneous vertebroplasty for spinal metastases.

We obtained clinical data from our database and categorized the patients into those who received rehabilitation treatment (R group) within one month and those who did not receive rehabilitation treatment (N group). The following variables were assessed: age, sex, primary tumor prognosis using the Katagiri score (slow/moderate/rapid), bone metastasis location (spinal, femoral/pelvic, humeral/rib), cancer treatments (chemotherapy and radiotherapy), ADL measured using the Barthel index (BI), and QOL assessed using the EuroQoL-5 Dimension (EQ-5D). First, the baseline characteristics and changes were compared between the two groups at one month. Propensity score matching was used to create comparable groups and was adjusted for potential differences in baseline characteristics for further analysis.

### 2.3 Rehabilitation protocol

Patients in the rehabilitation group received individualized physical therapy administered by licensed physical therapists. Rehabilitation sessions were conducted once daily, five days per week, with each session lasting approximately 20 min. The content and intensity of the intervention were adjusted according to each patient’s general condition, mobility, and tolerance. Importantly, the location and extent of bone metastases were carefully evaluated before initiating any physical intervention. Therapists developed customized programs to avoid mechanical stress on the metastatic lesions, and patients were thoroughly instructed on safe movement strategies to minimize the load on the affected sites. The rehabilitation intervention consisted of a multimodal approach including both aerobic and resistance exercises. Aerobic training included low-impact activities such as walking or cycling, while resistance training targeted major muscle groups in both the upper and lower extremities using bodyweight or elastic band exercises. The program primarily aimed to prevent pain and avoid overloading, and therapists closely monitored patients for signs of discomfort or the occurrence adverse events.

### 2.4 Statistical analysis

Propensity scores were calculated to balance the groups for several independent variables including age, sex, primary tumor prognosis, bone metastasis location, cancer treatment, and baseline BI. Two balanced groups were created using 1:1 matching. The Mann–Whitney *U* test was used for continuous variables, and the chi-square test was used for categorical variables to compare the two groups (R and N groups). Wilcoxon signed-rank test was used to compare the initial and second evaluations within each group. We performed multivariate logistic regression analysis (using the stepwise method with a likelihood ratio) to explore the independent factors associated with improvements in ADL and QOL in patients who underwent rehabilitation treatment. Statistical significance was set at *p* < 0.05 all analyses. Statistical analyses were performed using SPSS (version 19.0; IBM, Armonk, NY, USA).

## 3 Results

**Fig. 1:**
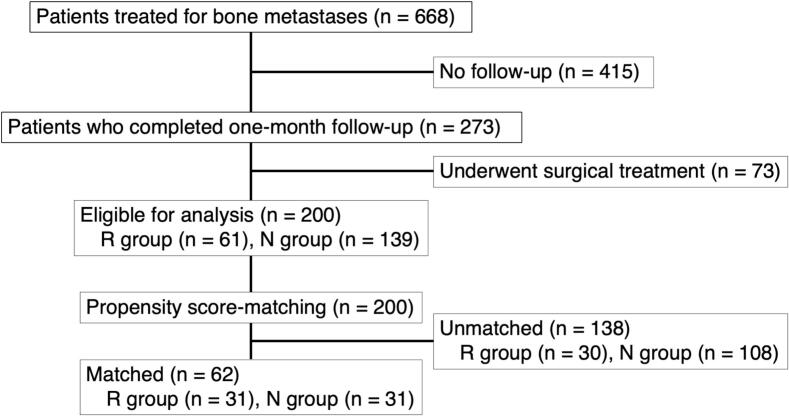
Flowchart of patient selection. R group: Patients who received rehabilitation treatment; N group: Patients who did not receive rehabilitation treatment.

A flowchart of the study is presented in Fig. 1. Data from 668 patients treated for bone metastases were retrospectively reviewed. Of these, 273 completed the one-month follow-up period. Among them, 200 who did not undergo surgical treatment for bone metastases during the study period were included in the final analysis. Propensity score matching resulted in 31 well-matched pairs for the final analyses.

Table 1 presents the baseline characteristics of the study cohort. Among patients with bone metastases, 30.5 % underwent rehabilitation. No serious adverse events related to the rehabilitation intervention such as pathological fractures or spinal cord paralysis were reported during the study period. Additionally, patients with a rapid primary lesion prognosis, as classified by Katagiri, were more likely to be in the N group (R group, 39.3 %; N group, 54.6 %; *p* = 0.053). No significant differences were observed between the two groups in terms of either bone metastasis location or chemotherapy and radiotherapy rates. However, the R group had a significantly lower baseline BI than the N group (R group: 66.8 ± 3.4, N group: 90.3 ± 1.6, *p* < 0.001). Similarly, baseline QOL scores were significantly lower in the R than in the N group (R group: 0.395 ± 0.038, N group: 0.644 ± 0.023, *p* < 0.001).

**Table 1:** Patient baseline characteristics.

All patients	R group (*n* = 61: 30.5 %)	N group (*n* = 139: 69.5 %)	*p*
Age (years)	73.1 ± 1.6	71.9 ± 1.1	0.548
Sex (male/female)	31/30	84/55	0.206
Primary lesions, as classified by Katagiri slow/moderate/rapid, n (%)	12/25/24 (19.7/41.0/39.3)	13/50/76 (9.4/36.0/54.6)	0.053
Bone metastasis			
Spinal metastasis, n (%)	50 (82.0)	120 (86.3)	0.426
Femoral and pelvic metastasis, n (%)	18 (29.5)	32 (23.0)	0.329
Upper arm and rib metastasis, n (%)	9 (14.8)	18 (12.9)	0.731
Treatment (chemotherapy, radiotherapy), n (%)			
(+, +)	12 (19.7)	20 (14.4)	0.443
(+, −)	15 (24.6)	35 (25.2)	
(−, +)	24 (39.3)	48 (34.5)	
(−, −)	10 (16.4)	36 (27.3)	
Barthel index at baseline	66.8 ± 3.4	90.3 ± 1.6	<0.001
EQ-5D at baseline	0.395 ± 0.038	0.644 ± 0.023	<0.001

The patient characteristics of each group after propensity score matching are shown in Table 2, with 31 patients in each group. No statistically significant differences were observed in baseline BI and EQ-5D between the two groups after propensity score matching (BI: 75.8 ± 4.2 vs. 77.9 ± 3.6; *p* = 0.797, EQ-5D: 0.458 ± 0.051 vs. 0.548 ± 0.043; *p* = 0.058). Other baseline characteristics were well balanced between the groups.

**Table 2:** Characteristics of the patients after propensity score matching.

All patients	R group (*n* = 31)	N group (*n* = 31)	*p*
Age (years)	75.9 ± 1.6	74.6 ± 1.9	0.905
Sex (male/female)	12/19	11/20	0.793
Primary lesions, as classified by Katagiri slow/moderate/rapid, n (%)	6/11/14 (19.4/35.5/45.1)	3/12/16 (9.7/38.7/51.6)	0.788
Bone metastasis			
Spinal metastasis, n (%)	26 (83.9)	30 (96.8)	0.198
Femoral and pelvic metastasis, n (%)	6 (19.4)	8 (25.8)	0.761
Upper arm and rib metastasis, n (%)	6 (19.4)	6 (19.4)	1.000
Treatment (chemotherapy, radiotherapy), n (%)			
(+, +)	8 (25.8)	6 (19.4)	0.915
(+, −)	3 (9.7)	6 (19.4)	
(−, +)	15 (48.4)	15 (48.4)	
(−, −)	5 (16.1)	4 (12.9)	
Barthel index at baseline	75.8 ± 4.2	77.9 ± 3.6	0.797
EQ-5D at baseline	0.458 ± 0.051	0.548 ± 0.043	0.058

**Fig. 2:**
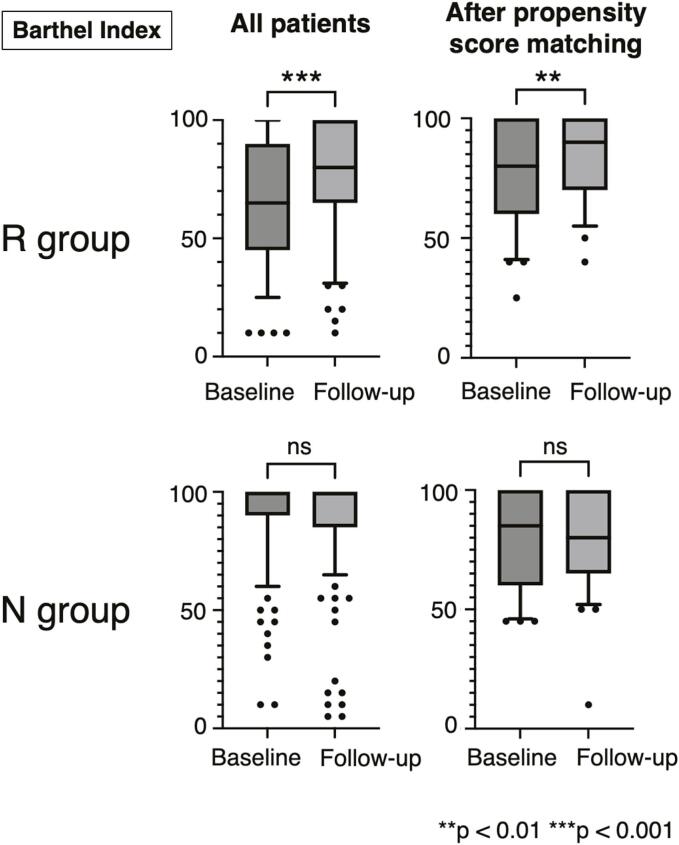
Changes in the Barthel index at baseline and follow-up in both groups, analyzed for the entire cohort and after propensity score matching. The central line in the box represents the median, the box indicates the interquartile range, the whiskers extend to the 10th–90th percentiles, and the dots represent outlier patients. R group: Patients who received rehabilitation treatment; N group: Patients who did not receive rehabilitation treatment; ns: Not significant.

Changes in the BI at baseline and follow-up for both groups are shown in Fig. 2, comparing the overall cohort and after propensity score matching. In the overall cohort, the R group showed a significant improvement in BI, with a baseline median of 65 (interquartile range [IQR]: 45–90) and a follow-up median of 80 (IQR: 65–100; *p* < 0.001). In contrast, no changes were observed in the N group. A similar tendency was observed after propensity score matching, with the R group showing a significant improvement in BI from a baseline median of 80 (IQR: 60–100) to a follow-up median of 90 (IQR: 70–100; *p* < 0.01).

**Fig. 3:**
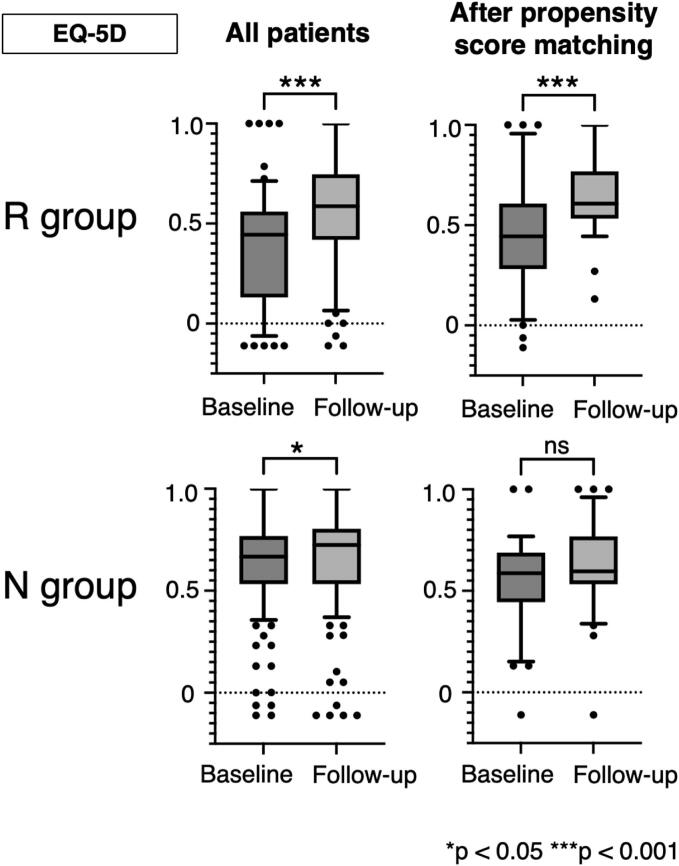
Changes in EuroQoL-5 Dimension (EQ-5D) at baseline and follow-up in both groups, analyzed for the entire cohort and after propensity score matching. The central line in the box represents the median, the box indicates the interquartile range, the whiskers extend to the 10th–90th percentiles, and the dots represent outlier patients. R group: Patients who received rehabilitation treatment; N group: Patients who did not receive rehabilitation treatment.

The changes in the EQ-5D scores at baseline and follow-up for both groups are shown in Fig. 3, comparing the overall and adjusted cohorts after propensity score matching. In the overall cohort, the R group showed a significant improvement in the EQ-5D, with a baseline median of 0.444 (IQR: 0.131–0.560) and a follow-up median of 0.587 (IQR: 0.419–0.747; *p* < 0.001). The N group also showed significant improvement, with a baseline median of 0.667 (IQR: 0.533–0.768) and a follow-up median of 0.724 (IQR: 0.533–0.804; *p* < 0.05). However, a significant improvement in the EQ-5D score was observed only in the R group after propensity score matching, with a baseline median of 0.444 (IQR: 0.282–0.608) and a follow-up median of 0.608 (IQR: 0.533–0.768; *p* < 0.001).

Finally, we performed a multivariate logistic regression analysis to identify factors influencing the improvement in BI and EQ-5D scores in patients with bone metastases who underwent rehabilitation treatment. The dependent variables were improvements in BI and EQ-5D, and the independent variables included sex, age, primary tumor prognosis using the Katagiri score, and the presence of chemotherapy, radiotherapy, spinal metastasis, femoral metastasis, or humeral/rib metastasis. Significant factors associated with BI improvement included chemotherapy (odds ratio: 4.03, 95 % confidence interval: 1.44–11.25, *p* = 0.008). Regarding EQ-5D, chemotherapy was also a significant factor influencing the improvement in QOL (odds ratio: 5.29, 95 % confidence interval: 1.89–14.79, *p* = 0.002).

## 4 Discussion

This study investigated the implementation of rehabilitation in patients with bone metastases who had not previously undergone surgery. Using propensity score matching, we demonstrated that rehabilitation significantly improved the ADL and QOL of life. Previous studies have reported that rehabilitation treatment for patients with spinal metastases improves survival outcomes [13] and exercise therapy for patients with prostate cancer and bone metastases effectively maintains physical function without adverse events [14]. However, these reports included patients who underwent surgical treatment for bone metastases, making our study the first to demonstrate the effectiveness of rehabilitation in patients with bone metastases who did not undergo surgery.

One of the most severe complications associated with bone metastasis are skeletal-related events (SREs), including pathological fractures that impair mobility, and vertebral compression fractures that may lead to spinal cord compression [3]. SREs significantly affect patients’ ADL and QOL and are closely associated with poor prognosis [15]. Shinoda et al. reported that preventing SREs could improve not only the QOL of patients with bone metastases but also their mental function and overall symptoms [16]. Given the risk of SREs, rehabilitation of patients with bone metastases should be carefully considered. Medical personnel with expertise in bone metastasis management should oversee rehabilitation to ensure appropriate risk management. It is essential to ensure that rehabilitation accounts for these risks as they can lead to adverse events and harm to the patient. Notably, in the present study, none of the patients developed pathological fractures or spinal cord paralysis during rehabilitation.

In this study, the rehabilitation implementation rate in patients with bone metastases was 30.5 %. Rehabilitation treatment was not provided to patients with bone metastases who were functionally independent of their ADL at baseline or to those with a poor prognosis according to the primary lesion classification. Although it is generally reasonable that patients with independent ADL may not require rehabilitation, all participants in this study had bone metastases, a condition that rarely heals. This patient population is at high risk of developing motor dysfunction and experience a decline in ADL over time [2]. Therefore, future studies are needed to explore whether rehabilitation interventions, including exercise therapy to prevent motor function deterioration and movement guidance to prevent SREs, can help maintain ADL levels and reduce the risk of SREs in functionally independent patients with bone metastases. At our institution, rehabilitation may be initiated through a referral to rehabilitation specialists from the primary department responsible for the management of cancer patients. However, the decision to request for rehabilitation is often left to the discretion of the primary department, which may affect the overall rehabilitation implementation rate. One potential strategy to improve this situation at our institution and those with similar protocols is the adoption of a multidisciplinary treatment approach that includes medical personnel with expertise in bone metastasis management [17]. In recent years, Bone Metastasis Cancer Boards (BMCBs) have been increasingly utilized as part of multidisciplinary care [12,18,19]. However, only 16 % of the designated cancer treatment centers reported holding BMCB meetings [20]. Although BMCB meetings were held at our institution during the study period, rehabilitation treatment rates remained low. This finding suggests that in addition to assessing indications for surgery or radiation therapy, BMCBs should more systematically involve rehabilitation physicians as core team members. Early and proactive participation may help identify candidates for preventive rehabilitation interventions and promote timely referral, even among patients who are functionally independent at baseline.

Finally, we identified chemotherapy as a key factor influencing improvements in ADL and QOL in patients with bone metastases who underwent rehabilitation. Fujii et al. reported that the absence of chemotherapy in patients with bone metastases contributes to a decline in ADL and QOL [12]. Similarly, Kobayashi et al. noted that patients with bone metastases admitted to rehabilitation wards, where chemotherapy is not feasible, often experience a decline in ADL [21]. Consistent with these findings, our results suggested that patients undergoing chemotherapy are more likely to benefit from rehabilitation therapy.

This study had some limitations. First, this was a single-arm retrospective analysis. Further multicenter, prospective, randomized controlled trials are required to confirm these findings. Second, the study included only patients who were available for follow-up at one month, and long-term outcomes were not assessed. Additionally, patients who could not be reassessed after one month due of transfer, home discharge, or death were excluded, which may indicate potential selection bias. Third, this study did not evaluate the timing of rehabilitation initiation. Therefore, the potential impact of early versus delayed rehabilitation remains unclear. Finally, although propensity score matching was performed, factors such as primary tumor history, prior treatments, and specific rehabilitation protocols varied among the cases, and their effects could not be fully adjusted. Despite these limitations, this study was the first to clarify the current state of rehabilitation treatment for patients with bone metastases and demonstrate its effectiveness. Given the clinical significance of these findings, further research is needed to optimize the rehabilitation strategies for this patient population.

## 5 Conclusion

This is the first study to investigate the implementation and effectiveness of rehabilitation in patients with bone metastases who have not undergone surgery. Our findings suggest that patients who received rehabilitation treatment showed significant improvements in ADL and QOL, with chemotherapy being the key associated factor. These results provide novel clinical insights into the role of rehabilitation in nonsurgical patients with bone metastases, a group for whom evidence-based treatment strategies remain scarce. Taken together, these results suggest that rehabilitation may be an effective therapeutic option for improving ADL and QOL in nonsurgically treated patients with bone metastases. Future prospective studies with larger cohorts are needed to clarify the indications, specific rehabilitation strategies, and overall effectiveness of rehabilitation treatment in this patient population.

## CRediT authorship contribution statement

**Ryo Yoshikawa:** Writing – original draft, Visualization, Project administration, Methodology, Investigation, Formal analysis, Data curation, Conceptualization. **Yasumitsu Fujii:** Writing – review & editing, Investigation, Formal analysis, Data curation. **Ryoga Kashima:** Writing – review & editing, Investigation, Formal analysis, Data curation. **Wataru Saho:** Investigation, Data curation. **Risa Harada:** Investigation, Data curation. **Daisuke Makiura:** Investigation, Data curation. **Katsuya Fujiwara:** Investigation, Data curation. **Junichiro Inoue:** Investigation, Data curation. **Yoshiki Takeoka:** Investigation, Data curation. **Ryoko Sawada:** Investigation, Data curation. **Naomasa Fukase:** Investigation, Data curation. **Keisuke Oe:** Investigation, Data curation. **Hitomi Hara:** Project administration, Investigation, Data curation. **Kenichiro Kakutani:** Project administration, Investigation, Data curation. **Toshihiro Akisue:** Project administration, Methodology, Conceptualization. **Yoshitada Sakai:** Writing – review & editing, Supervision, Software, Project administration, Methodology, Formal analysis, Conceptualization.

## Ethics approval and consent to participate

This study was approved by the Institutional Review Board of Kobe University Hospital (approval no. B210055). Written informed consent was obtained from all participants.

## Funding

This study did not receive any specific grants from funding agencies in the public, commercial, or non-profit sectors.

## Declaration of competing interest

The authors declare that they have no known competing financial interests or personal relationships that could have appeared to influence the work reported in this paper.

## References

[1] Huang J.-F., Shen J., Li X., Rengan R., Silvestris N., Wang M., Derosa L., Zheng X., Belli A., Zhang X.-L., Li Y.M., Wu A. (2020). Incidence of patients with bone metastases at diagnosis of solid tumors in adults: a large population-based study. Ann. Transl. Med..

[2] Seow H., Barbera L., Sutradhar R., Howell D., Dudgeon D., Atzema C., Liu Y., Husain A., Sussman J., Earle C. (2011). Trajectory of performance status and symptom scores for patients with cancer during the last six months of life. J. Clin. Oncol..

[3] Coleman R.E. (2001). Metastatic bone disease: clinical features, pathophysiology and treatment strategies. Cancer Treat. Rev..

[4] Doyle C., Kushi L.H., Byers T., Courneya K.S., Demark-Wahnefried W., Grant B., McTiernan A., Rock C.L., Thompson C., Gansler T., Andrews K.S. (2006). Nutrition, physical activity and cancer survivorship advisory committee, American cancer society, nutrition and physical activity during and after cancer treatment: an American Cancer Society guide for informed choices. CA Cancer J. Clin..

[5] Schmitz K.H., Courneya K.S., Matthews C., Demark-Wahnefried W., Galvão D.A., Pinto B.M., Irwin M.L., Wolin K.Y., Segal R.J., Lucia A., Schneider C.M., von Gruenigen V.E., Schwartz A.L. (2010). American college of sports medicine, american college of sports medicine roundtable on exercise guidelines for cancer survivors. Med. Sci. Sports Exerc..

[6] Rock C.L., Doyle C., Demark-Wahnefried W., Meyerhardt J., Courneya K.S., Schwartz A.L., Bandera E.V., Hamilton K.K., Grant B., McCullough M., Byers T., Gansler T. (2012). Nutrition and physical activity guidelines for cancer survivors. CA Cancer J. Clin..

[7] Stout N.L., Santa Mina D., Lyons K.D., Robb K., Silver J.K. (2021). A systematic review of rehabilitation and exercise recommendations in oncology guidelines, CA. Cancer J. Clin..

[8] Bunting R.W., Shea B. (2001). Bone metastasis and rehabilitation. Cancer.

[9] Saad F., Lipton A., Cook R., Chen Y.-M., Smith M., Coleman R. (2007). Pathologic fractures correlate with reduced survival in patients with malignant bone disease. Cancer.

[10] Hara H., Sakai Y., Kawamoto T., Fukase N., Kawakami Y., Takemori T., Fujiwara S., Kitayama K., Yahiro S., Miyamoto T., Kakutani K., Niikura T., Miyawaki D., Okada T., Sakashita A., Imamura Y., Sasaki R., Kizawa Y., Minami H., Matsumoto T., Matsushita T., Kuroda R., Akisue T. (2021). Surgical outcomes of metastatic bone tumors in the extremities (Surgical outcomes of bone metastases). J. Bone Oncol..

[11] Kanda Y., Kakutani K., Sakai Y., Yurube T., Miyazaki S., Takada T., Hoshino Y., Kuroda R. (2020). Prospective cohort study of surgical outcome for spinal metastases in patients aged 70 years or older. Bone Joint J..

[12] Fujii Y., Yoshikawa R., Kashima R., Saho W., Onishi H., Matsumoto T., Harada R., Takeoka Y., Sawada R., Fukase N., Hara H., Kakutani K., Akisue T., Sakai Y. (2024). Evaluation of changes in activities of daily living and quality of life of patients with bone metastasis who underwent conservative therapy through Bone Metastasis Cancer Boards. Medicina (Kaunas).

[13] Tang V., Harvey D., Park Dorsay J., Jiang S., Rathbone M.P. (2007). Prognostic indicators in metastatic spinal cord compression: using functional independence measure and Tokuhashi scale to optimize rehabilitation planning. Spinal Cord.

[14] Galvão D.A., Taaffe D.R., Spry N., Cormie P., Joseph D., Chambers S.K., Chee R., Peddle-Mcintyre C.J., Hart N.H., Baumann F.T., Denham J., Baker M., Newton R.U. (2018). Exercise preserves physical function in prostate cancer patients with bone metastases. Med. Sci. Sports Exerc..

[15] Coleman R.E. (2004). Bisphosphonates: clinical experience. Oncologist.

[16] Shinoda Y., Sawada R., Yoshikawa F., Oki T., Hirai T., Kobayashi H., Matsudaira K., Oka H., Tanaka S., Kawano H., Haga N. (2019). Factors related to the quality of life in patients with bone metastases. Clin. Exp. Metastasis.

[17] Vieillard M.-H., Thureau S. (2013). Multidisciplinary meetings dedicated to bone metastases: a historical perspective and rationale. Bull. Cancer.

[18] Miyazaki K., Kanda Y., Sakai Y., Yoshikawa R., Yurube T., Takeoka Y., Hara H., Akisue T., Kuroda R., Kakutani K. (2023). Effect of Bone metastasis cancer board on spinal surgery outcomes: a retrospective study. Medicina.

[19] Kashima R., Yoshikawa R., Saho W., Nakamura K., Tsuda Y., Harada R., Tatebayashi D., Sawada R., Kunihisa T., Sakai Y. (2024). Multidisciplinary treatment for breast cancer-related multiple bone metastases during pregnancy using Bone Metastasis Cancer Boards: a case report. Prog. Rehabil. Med..

[20] Morioka H., Kawano H., Takagi T., Abe S., Ogata N., Iwase S., Sakai Y., Oshima K., Ohe T., Nakamura K. (2023). Involvement of orthopaedic surgeons for cancer patients in orthopaedic training facilities certified by the Japanese Orthopaedic Association – a nationwide survey. J. Orthop. Sci..

[21] Kobayashi M., Yoshikawa R., Harada R., Date A., Kobayashi Y., Kozawa S., Sakai Y. (2022). Clinical outcome of patients with bone metastases in a convalescent rehabilitation ward: a case series of six patients. Prog. Rehabil. Med..

